# Reexamination of a Meta-Analysis of the Effect of Antioxidant Supplementation on Mortality and Health in Randomized Trials

**DOI:** 10.3390/nu2090929

**Published:** 2010-08-30

**Authors:** Hans K. Biesalski, Tilman Grune, Jana Tinz, Iris Zöllner, Jeffrey B. Blumberg

**Affiliations:** 1 Department of Biological Chemistry and Nutritional Science, University of Hohenheim, Garbenstrasse 28, D-70593 Stuttgart, Germany; Email: jana.tinz@uni-hohenheim.de; 2 Department of Nutritional Toxicology, Friedrich Schiller University Jena, Dornburger Strasse 24, D-07743 Jena, Germany; Email: tilman.grune@uni-jena.de; 3 Department of Epidemiology, Baden-Wuerttemberg State Health Office, Nordbahnhofstr. 135, D-70191 Stuttgart, Germany; Email: Iris.Zoellner@rps.bwl.de; 4 Jean Mayer USDA Human Nutrition Research Center on Aging, Tufts University, Boston, MA 02111, USA; Email: jeffrey.blumberg@tufts.edu

**Keywords:** antioxidants, supplementation, mortality, benefit/risk, meta-analysis

## Abstract

A recent meta-analysis of selected randomized clinical trials (RCTs), in which population groups of differing ages and health status were supplemented with various doses of β-carotene, vitamin A, and/or vitamin E, found that these interventions increased all-cause mortality. However, this meta-analysis did not consider the rationale of the constituent RCTs for antioxidant supplementation, none of which included mortality as a primary outcome. As the rationale for these trials was to test the hypothesis of a potential benefit of antioxidant supplementation, an alternative approach to a systematic evaluation of these RCTs would be to evaluate this outcome relative to the putative risk of greater total mortality. Thus, we examined these data based on the primary outcome of the 66 RCTs included in the meta-analysis via a decision analysis to identify whether the results provided a positive (*i.e.*, benefit), null or negative (*i.e.*, harm) outcome. Our evaluation indicated that of these RCTs, 24 had a positive outcome, 39 had a null outcome, and 3 had a negative outcome. We further categorized these interventions as primary (risk reduction in healthy populations) or secondary (slowing pathogenesis or preventing recurrent events and/or cause-specific mortality) prevention or therapeutic (treatment to improve quality of life, limit complications, and/or provide rehabilitation) studies, and determined positive outcomes in 8 of 20 primary prevention studies, 10 of 34 secondary prevention studies, and 6 out of 16 therapeutic studies. Seven of the eight RCTs with a positive outcome in primary prevention included participants in a population where malnutrition is frequently described. These results suggest that analyses of potential risks from antioxidant supplementation should be placed in the context of a benefit/risk ratio.

## 1. Introduction

Several research approaches, particularly observational studies, suggest that diets rich in antioxidant nutrients, particularly vitamins C and E, β-carotene, and selenium, and/or supplements containing one or more of these nutrients are associated with a reduction in the risk of several age-related chronic diseases, including some forms of cancer, cardiovascular, eye, and neurodegenerative diseases, e.g., references [1,2,3,4,5,6,7]. These relatively consistent results stimulated the initiation of several large scale, randomized clinical trials (RCTs), including the Nutrition Intervention Trials (NIT)[8]; Alpha-Tocopherol, Beta-Carotene Cancer Prevention (ATBC) study [9]; Gruppo Italiano per lo Studio della Sopravvivenza nell’Infarto miocardico-Prevenzione trial [10]; Heart Outcomes Prevention Study (HOPE) and Heart Outcomes Prevention Evaluation-The Ongoing Outcomes (HOPE-TOO)[11,12]; Supplementation in Vitamins and Mineral Antioxidants (SU.VI.MAX) study [13]; Women’s Health Study (WHS)[14]; Physicians’ Health Study (PHS) and Physicians’ Health Study II (PHS-II)[15,16]; Age-Related Eye Disease Study (AREDS)[17,18]; and Selenium and Vitamin E Cancer Prevention Trial (SELECT)[19]. The overall results of most of these RCTs have been presented as equivocal or null.

These RCTs were mostly designed to test the efficacy of antioxidant supplementation in the primary or secondary prevention of cancer and/or cardiovascular disease. Due largely to issues of cost and statistical power, most of these RCTs have tested only one or two antioxidant nutrients and, thus, may not have fully benefited from the dynamic interrelationships between these nutrient and other dietary and endogenous components of the antioxidant defense network. Importantly, none of these studies employed inclusion criteria such as low antioxidant nutrient intake or status and/or elevated biomarkers of oxidative stress, or determined outcome measures as to whether these parameters were affected by the intervention, elements necessary to testing the hypothesis that the putative benefit derived from these nutrients is due to their antioxidant actions [20,21]. 

The limitations of RCTs in testing the efficacy and safety of nutrients are not often fully appreciated. The RCT research approach was developed for drugs, where interventions are designed to cure a disease, not produced by their absence; nutrients prevent dysfunction that would result from their inadequate intake. Further, drug effects are generally intended to be acute, large, and with a specific target for action, while nutrient effects are typically chronic, modest, and polyvalent in scope. In addition, drug effects can be tested against a non-exposed (placebo) contrast group, whereas it is impossible and/or unethical to attempt a zero intake group for nutrients [22]. Nonetheless, RCTs are currently considered the “gold standard” for evaluating dietary interventions and, thus, receive the attention of most meta-analyses on this topic. 

In addition to using RCTs to test the potential health benefits of antioxidant supplements, meta-analyses may also provide an approach to determining their safety. Although specific adverse events have been reported for most nutrients, as reflected in established Tolerable Upper Intake Levels [23], all-cause or total mortality has sometimes utilized as a global indicator of safety in meta-analyses of RCTs [24,25,26]. In their meta-analysis, Bjelakovic *et al.* [27,28] concluded that vitamins A and E and β-carotene were associated with an increased risk of all-cause mortality with a relative risk (95% confidence interval) of 1.16 (1.10–1.24), 1.04 (1.01–1.07), and 1.07 (1.02–1.11), respectively. This report was based on 66 RCTs utilizing >1000 participants and excluded any study absent a report of mortality (747 articles) or considered by the authors to be of low methodologic quality (21 articles). The results from the RCTs selected by Bjelakovic *et al.* [27] may not be readily extrapolated to all other studies with low power due to too few subjects. Thus, we have selected a power-independent approach to reevaluate this meta-analysis with respect to its primary outcome of total mortality as well as examining their impact on the efficacy of the intervention.

## 2. Methods

We focus here on the primary endpoints of these RCTs. These data were extracted from all but two of the studies used in the Bjelakovic *et al.* [27] meta-analysis. The report by Chandra [29] (reference no. 39) has been excluded because of documented deficiencies in the integrity of data [30]. The article by Bonelli [31] (reference no. 57) has been excluded because a copy of this publication could not be obtained. The remaining 66 studies were reviewed and classified into three groups with separate analyses conducted for studies with primary prevention, secondary prevention, and therapeutic efficacy as their primary goal. Studies of primary prevention are defined as interventions in generally healthy people intended to reduce the risk of a disease or disability. Secondary prevention actions described here involve dietary interventions (nutrient supplementation) in a diagnosed group to slow the pathogenesis of the disease and/or prevent recurrent events or cause-specific mortality. Studies of therapeutic efficacy are directed to improving the quality of life, limiting complications, and/or providing rehabilitation to a patient group. Importantly, the therapeutic efficacy of dietary supplements is always tested concurrently with established standards of care, often including polypharmacy regimens, as withdrawing these other treatments would be unethical.

All the studies were independently classified by the authors and rated with regard to the primary study goal as: positive or goal achieved (+1), null outcome (0) or adverse effect (−1). Differences in classification or rating between the authors were documented and discussions held to resolve the conflicts until unanimous agreement was reached. The percentage of studies with positive results in the three groups of studies was compared using a χ^2^ test for homogeneity [32]. 

## 3. Results

### Efficacy of Antioxidant Interventions

Study outcomes were listed with regard to their design as primary or secondary prevention or therapy. When studies were reported to have more than one study outcome both were listed. Overall, 24 of 66 RCTs (36%) reported a positive outcome of the intervention indicating a benefit of antioxidant supplementation. Studies testing the effect of primary prevention by antioxidants showed positive outcomes in 8 of 21 studies (38%) while a single study reported a negative outcome. Reports from secondary prevention RCTs showed positive outcomes in 10 of 29 studies (34%) while 2 obtained negative outcomes. In studies directed to therapeutic outcomes, 6 of 16 RCTs (38%) achieved positive results. No significant difference (p = 0.78) was observed in secondary prevention trials regarding outcome compared with other studies. Most of these RCTs (60%) found neither benefit nor harm from the antioxidant supplement related to their primary goal (Table 1 and Table 2).

**Table 1:** Study outcome (intervention effects of antioxidant trials) with respect to primary goal and study type.

Group of Studies	Outcome	Number	Percent (%)
All studies (66 RCTs)	+1	24	36
	0	39	60
	−1	3	4
Primary prevention studies (20 RCTs)	+1	8	38
	0	12	57
	−1	1	5
Secondary prevention studies (34 RCTs)	+1	10	34
	0	17	59
	−1	2	7
Therapeutic intervention studies (16 RCTs)	+1	6	37.5
	0	10	62.5
	−1	0	0

Seven of the eight RCTs with a positive outcome in primary prevention included participants in a population where malnutrition is frequently described [8,13,33,34,35,36,37]. For example, five of these studies were conducted in the elderly, a population often described as at risk of micronutrient deficiencies [33,34,35,36,37]. The positive outcome in the SU.VI.MAX study of Hercberg *et al.* [13] was obtained only in males who, compared to the female participants, presented with a lower baseline status of antioxidants during enrolment into the study. The participants in the Nutrition Intervention Trials were residents of Linxian, China, an area with a high prevalence of malnutrition [8].

**Table 2:** Studies of Bjelakovic *et al.* [27] and their outcome with respect to the study type.

Ref. No. *	Author(s) [Ref. No.]	Publication Title	Outcome			Type of Study		
			+1	0	−1	Primary Prevention	Secondary Prevention	Therapy
35	Gillilan *et al.* [38]	Quantitative evaluation of vitamin E in the treatment of angina pectoris		x				x
36	McKeown-Eyssen *et al.* [39]	A randomized trial of vitamins C and E in the prevention of recurrence of colorectal polyps	x				x	
37	Greenberg *et al.* [40]	A clinical trial of beta carotene to prevent basal-cell and squamous-cell cancers of the skin. The Skin Cancer Prevention Study Group		x			x	
38	Penn *et al.* [36]	The effect of dietary supplementation with vitamins A, C and E on cell-mediated immune function in elderly long-stay patients: A randomized controlled trial	x			x		
40	Murphy *et al.* [41]	Impact of vitamin A supplementation on the incidence of infection in elderly nursing-home residents: A randomized controlled trial		x		x		
41	Blot *et al.* [8]	Nutrition intervention trials in Linxian, China: Supplementation with specific vitamin/mineral combinations, cancer incidence, and disease-specific mortality in the general population	x			x		
42	Li *et al.* [42]	Nutrition intervention trials in Linxian, China: Multiple vitamin/mineral supplementation, cancer incidence, and disease-specific mortality among adults with esophageal dysplasia		x			x	
43	Wenzel *et al.* [43]	Alcohol-induced toxic hepatitis-a "free radical" associated disease. Lowering fatality by adjuvant antioxidant therapy	x					x
44	Greenberg *et al.* [44]	A clinical trial of antioxidant vitamins to prevent colorectal adenoma. Polyp Prevention Study Group		x			x	
45	Pike and Chandra [37]	Effect of vitamin and trace element supplementation on immune indices in healthy elderly	x			x		
46	Takamatsu *et al.* [45]	Effects on health of dietary supplementation with 100 mg d-alpha-tocopheryl acetate, daily for 6 years	x			x		
47	de la Maza *et al.* [46]	Effects of long-term vitamin E supplementation in alcoholic cirrhotics		x				x
48	ter Riet *et al.* [47]	Randomized clinical trial of ascorbic acid in the treatment of pressure ulcers		x				x
49	Clark *et al.* [48]	Effects of selenium supplementation for cancer prevention in patients with carcinoma of the skin: A randomized controlled trial	x				x	
50	Hennekens *et al.* [15]	Lack of the effect of long-term supplementation with beta carotene on the incidence of malignant neoplasms and cardiovascular disease		x		x		
51	Hogarth *et al.* [49]	Nutritional supplementation in elderly medical in-patients: A double-blind placebo-controlled trial		x			x	
52	Richer [50]	Multicenter ophthalmic and nutritional age-related macular degeneration study, II: Antioxidant intervention and conclusions	x					x
53	Stephens *et al.* [51]	Randomised controlled trial of vitamin E in patients with coronary disease: Cambridge Heart Antioxidant Study (CHAOS)	x					x
54	Girodon *et al.* [33]	Effect of micronutrient supplementation on infection in institutionalized elderly subjects: A controlled trial	x			x		
55	Moon *et al.* [52]	Effect of retinol in preventing squamous cell skin cancer in moderate-risk subjects: A randomized, double-blind, controlled trial	x				x	
56	Sano *et al.* [53]	A controlled trial of selegiline, alpha-tocopherol, or both as treatment for Alzheimer`s disease: The Alzheimer`s Disease Cooperative Study	x					X
58	GISSI [10]	Dietary supplementation with N-3 polyunsaturated fatty acids and vitamin E after myocardial infarction: Results of the GISSI-Prevenzione trial		x			x	
59	Girodon *et al.* [34]	Impact of trace elements and vitamin supplementation on immunity and infections in institutionalized elderly patients: A randomized controlled trial	x			x		
60	Green *et al.* [54]	Daily sunscreen application and beta-carotene supplementation in prevention of basal-cell and squamous-cell carcinomas of the skin: A randomised controlled trial		x		x		
61	Boaz *et al.* [55]	Secondary prevention with antioxidants of cardiovascular disease in endstage renal disease (SPACE): Randomised placebo-controlled trial	x				x	
62	Correa *et al.* [56]	Chemoprevention of gastric dysplasia: Randomized trial of antioxidant supplements and anti-helicobacter pylori therapy	x				x	
63	Jacobson *et al.* [57]	Effects of a 6-month vitamin intervention on DNA damage in heavy smokers		x		x		
64	Age-Related Eye Disease Study research Group [18]	A randomized, placebo-controlled, clinical trail of high-dose supplementation with vitamins C and E and beta carotene for age-related cataract and vision loss		x			x	
65	Brown *et al.* [58]	Simvastatin and niacin, antioxidant vitamins, or the combination for the prevention of coronary disease		x			x	
66	Desnuelle *et al.* [59]	A double-blind, placebo-controlled randomized clinical trail of alpha-tocopherol (vitamin E) in the treatment of amyotrophic lateral sclerosis	x					x
67	Stevic *et al.* [60]	A controlled trial of combination of methionine and antioxidants in ALS patients	x					x
68	You *et al.* [61]	An intervention trial to inhibit the progression of precancerous gastric lesions: Compliance, serum micronutrients and S-allyl cysteine levels, and toxicity		x			x	
69	de Gaetano and Collaborative Group of the Primary Prevention Project [62]	Low-dose aspirin and vitamin E in people at cardiovascular risk: A randomised trial in general practice		x			x	
70	de Waart *et al.* [63]	Effect of glutathione S-transferase M 1 genotype on progression of atherosclerosis in lifelong male smokers		x			x	
71	Chylack *et al.* [64]	The Roche European American Cataract Trial (REACT): A randomized clinical trial to investigate the efficacy of an oral antioxidant micronutrient mixture to slow progression of age-related cataract	x				x	
72	Graat *et al.* [65]	Effect of daily vitamin E and multivitamin-mineral supplementation on acute respiratory tract infections in elderly persons: A randomized controlled trial			x	x		
73	Heart Protection Study Collaborative Group [66]	MRC/BHF Heart Protection Study of antioxidant vitamin supplementation in 20536 high-risk individuals: A randomised placebo-controlled trial		x			x	
74	Hodis *et al.* [67]	Alpha-tocopherol supplementation in healthy individuals reduces low-density lipoprotein oxidation but not atherosclerosis		x		x		

75	Waters *et al.* [68]	Effects of hormone replacement therapy and antioxidant vitamin supplements on coronary atherosclerosis in postmenopausal women: A randomized controlled trial			x		x	
76	White *et al.* [69]	Dietary antioxidants and DNA damage in patients on long-term acid suppression therapy. a randomized controlled study		x			x	
77	Wluka *et al.* [70]	Supplementary vitamin E does not affect the loss of cartilage volume in knee osteoarthritis: A randomized placebo controlled study		x				x
78	Collins *et al.* [71]	PoleStriding exercise and vitamin E for management of peripheral vascular disease		x				X
79	Prince *et al.* [72]	Oral antioxidant supplementation for fatigue associated with primary biliary cirrhosis: Results of a multicentre, randomized, placebo-controlled, cross-over trial		x				x
80	Salonen *et al.* [73]	Antioxidant supplementation in atherosclerosis prevention study: Six-year effect of combined vitamin C and E supplementation on atherosclerotic progression: The Antioxidant Supplementation in Atherosclerosis Prevention (ASAP) Study	x				x	
81	Sasazuki *et al.* [74]	The effect of 5-year vitamin C supplementation on serum pepsinogen level and Helicobacter pylori infection	x				x	
82	Takagi *et al.* [75]	Pilot clinical trial of the use of alpha-tocopherol for the prevention of hepatocellular carcinoma in patients with liver cirrhosis		x			x	
83	Virtamo *et al.* [9]	Incidence of cancer and mortality following alpha-tocopherol and beta-carotene supplementation: A postintervention follow-up		x		x		
84	Allsup *et al.* [76]	Can a short period of micronutrient supplementation in older institutionalized people improve response to influenza vaccine?		x		x		
85	Goodman *et al.* [77]	The Beta-Carotene and Retinol Efficacy Trial: Incidence of lung cancer and cardiovascular disease mortality during 6-year follow-up after stopping beta-carotene and retinol supplements		x		x		
86	Hercberg *et al.* [13]	The SU.VI.MAX Study: A randomized, placebo-controlled trial of the health effects of antioxidant vitamins and minerals	x			x		
87	Manuel-y-Keenoy *et al.* [78]	Impact of vitamin E supplementation on lipoprotein peroxidation and composition in type 1 diabetic patients treated with atorvastatin		x			x	
88	McNeil *et al.* [79]	Vitamin E supplementation and cataract: Randomized controlled trial		x			x	
89	Meydani *et al.* [35]	Vitamin E and respiratory tract infections in elderly nursing home residents: A randomized controlled trial	x			x		
90	Mezey *et al.* [80]	A randomized placebo controlled trial of vitamin E for alcoholic hepatitis		x				x
91	Richer [81]	Double-masked, placebo-controlled, randomized trial of lutein and antioxidant supplementation in the intervention of atrophic age-related macular degeneration: The Veterans LAST study (Lutein Antioxidant Supplementation Trial)	x				x	
92	Avenell *et al.* [82]	Effect of multivitamin and multimineral supplements on morbidity from infections in older people (MAVIS trial): Pragmatic, randomised, double blind, placebo controlled trial		x		x		
93	Graf *et al.* [83]	High dose vitamin E therapy in amyotrophic lateral sclerosis as add-on therapy to riluzole: Results of a placebo-controlled double-blind study		x				x
94	Lee *et al.* [14]	Vitamin E in the primary prevention of cardiovascular disease and cancer: The Women`s Health Study: A randomized controlled trial		x		x		
95	Limburg *et al.* [84]	Randomized, placebo-controlled, esophageal squamous cell cancer chemoprevention trial of selenomethionine and celecoxib		x			x	
96	Lonn *et al.* [12]	Effects of long-term vitamin E supplementation on cardiovascular events and cancer: A randomized controlled trial			x		x	
97	Marras *et al.* [85]	Parkinson Study Group. Survival in Parkinson disease: Thirteen-year follow-up of the DATATOP cohort		x				x
98	Mooney *et al.* [86]	Antioxidant vitamin supplementation reduces benzo(a)pyrene-DNA adducts and potential cancer risk in female smokers		x		x		
99	Petersen *et al.* [87]	Vitamin E and donepezil for the treatment of mild cognitive impairment		x				x
100	Tam *et al.* [88]	Effects of vitamin C and E on oxidative stress markers and endothelial function in patients with systemic lupus erythematosus: A double blind, placebo controlled pilot study		x			x	
101	Witte *et al.* [89]	The effect of micronutrient supplementation on quality-of-life and left ventricular function in elderly patients with chronic heart failure	x				x	
102	Rayman *et al.* [90]	Impact of selenium on mood and quality of life: A randomized, controlled trial		x		x		

* The numbering of the references is consistent with those reported in Bjelakovic *et al.* [27].

## 4. Discussion

Assessing the primary goal (*i.e.*, the determination of benefit) of the RCTs analyzed by Bjelakovic *et al.* [27] places their conclusion about the risk of mortality in a context relevant to the consideration of using antioxidant supplements in health promotion and therapeutic treatments. Clearly, increases in disease-specific or all-cause mortality are never objectives of intervention studies but, when observed, are a reason to stop the protocol early. Nonetheless, it is worth noting that the risk of mortality in any RCTs of nutrients will be substantially dependent upon the nature of the cohort, including parameters such as advanced age, severe disease status, toxicity of drug treatments, *etc*. Importantly, in our analysis, we did not evaluate as positive, null or adverse the outcomes of the 405 studies excluded from the Bjelakovic *et al.* [27] study because no death occurred. This is a limitation to our study as it does not provide a full assessment of the potential benefits versus the risk of total mortality from antioxidant supplementation.

With regard to the efficacy of antioxidant supplementation in the RCTs included in the meta-analysis by Bjelakovic *et al.* [27], we find that the benefit of the intervention was statistically significant principally in those populations generally characterized at risk for micronutrient deficiencies, including those of vitamins C and E, selenium, and beta-carotene as well as other nutrients such as zinc that contribute to the antioxidant defense network. This relationship may suggest that dietary supplementation for the prevention or treatment of chronic diseases is likely to be most effective in those with inadequate intakes, though absent overt deficiency syndromes. Further, this relationship also suggests there is a threshold nutrient status above which additional intake via supplementation might provide no further benefit. However, the threshold for adequate intake of any nutrient is recognized as dependent on an individual’s specific requirements as affected by parameters such as age, sex, health status, and nutrigenomic factors such as polymorphisms. The contribution of polymorphisms to antioxidant defenses can be illustrated by the manganese-dependent superoxide dismutase polymorphism associated with a lower risk of prostate cancer in the presence of high intakes of selenium to optimize glutathione peroxidase activity [91].

Critical to the validity of a meta-analysis is the statistical accounting for study variability, particularly when information from multiple RCTs is combined, including the requirement for considering model uncertainty and trial effect. For example, Berry *et al.* [92] employed a Bayesian hierarchical meta-analytic method rather than the frequentist approach to synthesize results from the RCTs of vitamin E supplementation used by Bjelakovic *et al.* [27] and Miller *et al.* [26] and concluded that vitamin E intake is unlikely to affect mortality regardless of dose. Other criticisms of the Bjelakovic *et al.* [27] meta-analysis have been noted, such as including small RCTs with few deaths; attributing deaths occurring after only a few months of antioxidant treatment; combining different nutrients in different forms with a large range of doses in a wide variety of population groups with varying health status; misclassifying some studies as being of high risk of bias; and basing the conclusion about antioxidant-specific effects on models that excluded selenium trials. However, no study before has considered evaluating the same studies selected by Bjelakovic *et al.* [27] for their benefits. 

Our approach to considering overall beneficial outcomes of the RCTs selected by Bjelakovic *et al.* [27] provides a balancing perspective to their meta-analysis focused exclusively on the risk of all-cause mortality. It is worthwhile noting limitations associated with the use of meta-analyses of total mortality as an indication of harm without determining the cause of death. Bjelakovic *et al.* [26] did not investigate cause-specific mortality and thus could not eliminate those that lack any biological plausibility to antioxidant (or pro-oxidant) toxicity such as accidental deaths and homicides. 

It should be noted that we did not include in our evaluation benefits found only in subgroups from the RCTs selected by Bjelakovic *et al.* [27]. For examples, we judged the WHS to have an overall null outcome among its 39,876 women >45 years of age receiving a 600 IU vitamin E supplement every other day for 10 years despite the observation by Lee *et al.* [14] of a significant reduction in the relative risk (RR) of the primary outcome of myocardial infarction, cardiovascular death or stroke among women >65 years of age (RR = 0.74; 95% Confidence Interval [CI]: 0.59–0.93; P = 0.009). Similarly, we judged the Vitamin E Atherosclerosis Study (VEAPS) to have a null effect (P = 0.08) on its primary trial endpoint of the rate of change in right distal common carotid artery intima-media thickness in 332 healthy men and women ≥40 years receiving 400 IU vitamin E for three years despite the observation by Hodis *et al.* [67] of significantly reduced concentrations of circulating oxidized low density lipoprotein (LDL) and increased resistance of LDL to oxidation, both biomarkers of cardiovascular disease risk. While such subgroup analyses typically lack the statistical power from which to draw definitive conclusions, these results do suggest that further RCTs are warranted to test the potential benefit of the intervention in that population. 

As the relative number of positive outcomes evaluated in our analysis was based only on those studies analyzed by Bjelakovic *et al.* [27], it is possible we have underestimated the proportion of studies demonstrating benefit. For example, Bjelakovic *et al.* [27] selected the mortality data only from the AREDS study [18] report examining the effect of a combination antioxidant supplement on the development of cataracts and vision loss which had a null outcome (Table 2). However, the same mortality data, albeit provided in more detail, is available from the AREDS study [17] report examining the effect of the same supplement on age-related macular degeneration and vision loss in which a significant positive outcome was achieved. Further, in evaluating the positive, null and adverse outcomes of this set of studies, we did not take advantage of follow-up reports published after publication of the Bjelakovic *et al.* [27] article. For example, Watters *et al.* [93] recently examined prospectively the serum antioxidant status in men diagnosed with prostate cancer during the ATBC Study of 29,133 Finish smokers and in their 20 years follow-up found improved survival among the men with higher serum α-tocopherol at baseline (Hazard Ratio [HR] = 0.67; 95% CI: 0.45–1.00; P = 0.03), especially among cases who had received the vitamin E supplement and who were in the highest quintile of α-tocopherol at baseline (HR = 0.51; 95% CI: 0.20–0.90; P = 0.04) and at the 3-year follow-up measurement (HR = 0.26; 95% CI, 0.09–0.71; P = 0.02). Thus, a review including follow-up of earlier RCTs and new RCTs, e.g., of the SELECT [19] would offer a more comprehensive consideration of the benefit in relation to a potential (mortality) risk of antioxidant supplementation than provided here.

As noted above, the outcome of human studies on dietary antioxidants will depend on the initial status of the antioxidant defense network and oxidative stress in each subject, the dose(s) of the nutrient(s), and the concentration threshold for action of each nutrient. One of the major challenges in conducting RCTs to test the efficacy and safety of antioxidants (and other nutrients) as dietary supplements in reducing the risk of chronic disease, especially in primary prevention, is the need for very long durations of the intervention as we lack validated intermediary biomarkers of these conditions. The RCT conducted by Milman *et al.* [94] demonstrating the reduction of cardiovascular events (myocardial infarct, cardiovascular death, and stroke) by 400 IU/d vitamin E in type 2 diabetics with a haptoglobin 2-2 genotype but not other alleles suggests as well the need to consider nutrient/gene interactions in the study design. Total deaths within a single study or as part of a meta-analysis may be a utilized as one measure of the toxicity of a chronic intervention; however, collection of specific mortality data is essential as RCTs of dietary supplements cannot otherwise establish either biological plausibility or causality due to the inability to determine the response to discontinuation and the response to rechallenge.

The operational definition of dietary antioxidants provided by the U.S. Institute of Medicine states that these nutrients significantly decrease the adverse effects of reactive species, such as reactive oxygen and nitrogen species, on normal physiological function in humans [95]. These adverse effects are essentially described as oxidatively modified products of DNA, lipids, and protein such as 8-hydroxy-2’-deoxyguanosine, F_2__α_-isoprostanes, and protein carbonyls, respectively. However, the efficacy of different antioxidants in achieving this effect is critically dependent on a variety of factors, including the baseline value of these biomarkers and the dose and duration of treatment. While lowering these biomarkers has been associated in many experimental and observational studies with a reduced risk of some age-related pathologies and chronic diseases, no evidence appears available to suggest they are correlated with premature mortality. Importantly, the classification of vitamins C and E, carotenoids, flavonoids and related polyphenols as dietary antioxidants fails suggest their other various mechanisms of action, including anti-inflammation, induction of phase 2 detoxification enzymes, and modulation of redox sensitive signal transduction and gene expression. Thus, new studies of the clinical efficacy and safety of antioxidants as dietary supplements must consider appropriate forms and doses of the ingredients, a duration relevant to the pathogenesis and/or progression of the disease, and the biologically relevant molecular targets in populations most likely to respond in a fashion providing a high benefit/risk ratio. 
